# In Vivo Bioconcentration, Distribution and Metabolization of Benzophenone-3 (BP-3) by *Cyprinus carpio* (European Carp)

**DOI:** 10.3390/foods11111627

**Published:** 2022-05-31

**Authors:** Florentina Laura Chiriac, Irina Eugenia Lucaciu, Iuliana Paun, Florinela Pirvu, Stefania Gheorghe

**Affiliations:** National Research and Development Institute for Industrial Ecology—ECOIND, Drumul Podu Dambovitei 57–73, Sector 6, 060652 Bucharest, Romania; laura.chiriac@incdecoind.ro (F.L.C.); irina.lucaciu@incdecoind.ro (I.E.L.); iuliana.paun@incdecoind.ro (I.P.); florinela_pirvu@yahoo.com (F.P.)

**Keywords:** benzophenone-3, *Cyprinus carpio*, bioconcentration, metabolites

## Abstract

Organic UV-filters, such as oxybenzone (BP-3), have attracted researcher attention in recent years due to its capacity to interfere with the proper functioning of the endocrine system and its widespread presence in the aquatic environment. The aim of this study was to investigate the bioconcentration, distribution and metabolization of BP-3 in one of the most common fish species in Romania, namely *Cyprinus carp* (European carp). Exposure experiments were performed for 11 weeks using a BP-3 nominal concentration level of 100 µg/L. The BP-3 concentration level decreased over time and needed to be re-established daily. Biological samples (fish organs and tissues) from control and test were taken at t0 (before contamination) and at t3, t5, t8 and t11 weeks from the beginning of the experiment. From the third week, BP-3 was identified and quantified, in all organs, in concentration values ranging between 3.2 and 782 ng/g d.w., the highest concentration being detected in the intestinal content, followed by gonads (up to 468 ng/g d.w.) and skin (up to 453 ng/g d.w.). In the case of gill and liver, the BP-3 concentration increased in the first five weeks, and then decreased to 15 and 6 ng/g d.w., respectively, which could be explained by a fast BP-3 metabolization. During the exposure period, six metabolites were identified through LC-MS/MS, all of them known for their endocrine disruptor and toxic properties being higher than those of the parent compound. The study is important from an ecological perspective and also in view of human health concerns involving food quality.

## 1. Introduction

Environmental concerns regarding the occurrence of organic ultraviolet (UV)-filters in aquatic ecosystems are due to the harmful effects of these micropollutants to humans and wildlife, and to the fact that thousands of tons of sunscreen are ending up in aquatic environments every year [1,2]. All these chemical substances are known for their developmental toxicity and endocrine disruption properties [3]. One of the most used UV-filter compounds is Oxybenzone or Bezophenone-3 (BP-3), classified as active ingredient in cosmetics and in human drugs as UV-filter. BP-3 is an organic UV-filter used in sunscreens and other cosmetic products to protect skin, hair and lips against UV radiation, but also in all kinds of industrial products as additive to enhance physical performance and durability [4]. According to Globally Harmonized System (GHS), BP-3 is classified as very toxic for aquatic life (H400) and toxic to aquatic life with long lasting effects (H411), based on algae (EC_50_ = 0.67 mg/L) and daphnia toxicity tests (EC_50_ = 1.87 mg/L). BP-3 showed no inhibitory effect on microorganisms of activated sludge with a NOEC of 100 mg/L. The freshwater fish short term toxicity tests showed LC_50_=3.8 mg/L, NOEC = 0.72 mg/L and LOEC =1.05 mg/L based on mortality effect [5]. PubChem Database present ecotoxicological information of BP-3 as follows: *Isochrysis galbana* (Haptophyte) EC_50_ (72h) = 13.87 µg/L (decreased population growth rate) [6]; *Acropora cervicornis* (Staghorn Coral) larva LC_50_ (4h) = 9 µg/L [7]; *Mytilus galloprovincialis* (Mediterranean Mussel) egg EC_50_ (48 h) = 3472.59 µg/L (decreased normal development) [6]; *Dugesia japonica* (Flatworm) LC_50_ (24 h) = 2200 µg/L [8]. The experiments performed with fish (*Betta splendens*), 10–1000 µg/L BP-3 for 28 days, demonstrate that this substance can induce endocrine disrupting activity in male fish [9]. Endocrine balance and reproduction performance in Japanese medaka fish (*Oryzias latipes*) could be affected by exposure to 4.7–90 µg/L BP-3 for 14 days [10]. Other studies have reported that BP-3 alters hormones, inducing vitellogenesis and generation of free radicals and changes in antioxidant enzymes in Zebrafish eluthero-embryos exposed to 1–1000 µg/L of BP-3 [11]. Both direct and indirect activities, such as swimming and wastewater treatment plant effluents, are mainly responsible for the high concentration levels of BP-3 and other organic UV-filters in aquatic ecosystems [12]. Thus, BP-3 is a ubiquitous presence in wastewater, surface water, seawater, sediments, biota and even in humans all over the world [13,14,15,16,17,18,19]. Indeed, BP-3 was found in surface water in various concentration domains: 2–35 ng/L (Swiss lakes, 2002) [4]; 2.6–88, 40–83, 12–79, 83–101 and 28–44,000 ng/L (Antarctic, Germany, Spain, Taiwan and the UK, 1996–2016) [20,21]; 15–36 ng/L (UK) [22].

In a recent study performed in Gran Canaria Island (Spain) on local wastewater treatment plants (WWTPs), BP-3 was the most detected compound in the seawater samples (frequency detection of 83%) and also in wastewater influents and effluents [23]. Although BP-3 is frequently detected in fresh and marine environments, both in water and biota, the bioaccumulation and biotransformation processes of BP-3 in fish has barely been studied. In recent years, a few studies focused their research activities on understanding the bioaccumulation and effects of BP-3 or other organic UV-filters in various aquatic organisms under laboratory condition: mussels [24,25], corals [2,26], fish [27,28] and swamp crayfish [29]. Two ways of contamination were approached, waterborne or dietary, using an exposed concentration range of 1–1000 μg/L. The estimated hazard for aquatic organisms showed the following PNEC values (Predicted No Effect Concentration): freshwater—6.7 µg/L (assessment factor 1000); marine water—0.067 µg/L (assessment factor 1000); sediment freshwater—0.066 mg/kg d.w. marine sediment—0.007 mg/kg d.w. With a log Kow of 3.45, the BP-3 bioaccumulation potential in fish is estimated to be low [5]. Other studies reported BCFs of 39–160 and 33–156 in fish for BP-3, using carp (*Cyprinus carpio*) which were exposed over a 10-week period at respective concentrations of 0.1 and 0.01 mg/L. The results suggest that BP-3 bioconcentration in aquatic organisms is moderate to high [30,31]. Fish showed significantly higher accumulation of the UV-filter compounds than the invertebrates. The concentrations of BP-3 and other UV organic compounds were markedly higher in the detritus of feeding fishes than of carnivorous and planktonic fishes, suggesting that bioaccumulation depends on the dietary habits of the organisms [32,33]. Furthermore, studies on the transformation and metabolism of BP-3 in organs have been shown to be of particular importance for metabolomics to ensure that the effects and concentration of anthropogenic by-products are not underestimated, as they may be even more toxic than the parent compound [34,35]. However, the number of studies conducted in this research direction is extremely low, and the lack of information makes it difficult to understand the bioaccumulation and biotransformation mechanism of BP-3, especially in fish, in real ecosystems. The need to understand comes from the recognition that fish are an important source of food, and their consumption can harm the health of both aquatic organisms located higher in the food chain and humans.

In this context, the objectives of the present study were to evaluate: (i) the uptake and distribution of BP-3 in fish organs (brain, gonad, intestine and intestinal contents, kidneys, muscles, liver, gills, skin and scaly) and (ii) the BP-3 metabolization in fish organs using liquid chromatography with tandem mass spectrometry (LC-MS/MS) for detection and quantification of chemical concentration levels.

## 2. Results and Discussion

### 2.1. Dilution Water and Testing Solution Characteristics

The dilution water quality corresponds to OECD standard testing conditions: pH 7.4 ± 1.5, total hardness 140 ± 5 mgCaCO_3_/L, dissolved oxygen 7.93 ± 1.50 mgO_2_/L, temperature 22 ± 2 °C, conductivity 361 ± 10 µS/cm, chemical oxygen demand (COD) 10 ± 0.02 mg/L, suspensions 5 ± 0.5 mg/L, free residual chlorine <0.03 mg/L, no heavy metals or other harmful compounds are detected. In the testing solutions with BP-3, the following conditions were reported: pH 7.68 ± 0.38, dissolved oxygen 6.42 ± 1.51 mgO_2_/L, temperature 22.5 ± 1 °C, conductivity 301 ± 31.38 µS/cm, COD 54–386 mgO_2_/L, suspensions 8–40.8 mg/L, and total hardness 138 ± 5 mgCaCO_3_/L. Organic content and suspensions varied before solutions renewal at 48 h, before removal of unconsumed or undigested waste.

### 2.2. Stability of BP-3 in Water

A preliminary experiment was performed, consisting in the evaluation of BP-3 stability in water solution. The aquarium with the same conditions as the one with the exposure experiment, but without fish, was purposefully contaminated with BP-3 solution so that the resulting nominal concentration was 100 µg/L. BP-3 showed a very good stability, with a decrease after 120 h of only 1.52% (Table 1).

### 2.3. BP-3 Water Concentrations

Regarding the fish exposure experiments, water samples from the exposure aquarium were taken after 1 h from BP-3 contamination and analyzed by LC-MS / MS to determine the exact concentration of BP-3 in the aquarium. A 20% decrease in baseline was observed immediately after adding the fish to the aquarium (Table 2). After 24 h from the beginning of the test, the concentration of BP-3 decreased drastically, resulting in less than 10% of the initial concentration in the aquarium being found. Subsequently, the solution in the aquarium was spiked with BP-3 stock solution, to reach the theoretical concentration of 100 µg/L. It was observed that, even when samples were taken immediately after contamination, the concentration of organic compound did not reach the desired concentration of 100 µg/L. Moreover, taking water from the test aquarium at 2 h after contamination, a decrease in BP-3 of up to 20% was observed.

After 9 days of exposure, the variation in BP-3 concentration measured in the morning samples was relatively low, ranging from 55–70 µg/L (Supplementary Materials, Figure S2). The same trend was observed throughout the experiment, with lower concentrations being observed in the water samples analyzed after the weekend. After about 70 days, the concentration of BP-3 began to stabilize and remain constant, which was an indication of the reaching of the equilibrium phase of BP-3 in fish. Similar variations of BP-3 concentration levels during the experiments were also reported in similar studies [26,27].

### 2.4. Exposed Fish Observations

At the end of the test (t_11_), the BP-3-exposed fish were visually analyzed, measured and weighed. The results were compared to the initial data (t_0_). The final values are presented in Table S6. The biometric indices remain constant compared to the initial values both in the case of the control and the test. An increase in weight of 8% was found in the test fish compared to the control test. There were no mortalities reported during the exposure period and no deviant behaviors.

### 2.5. BP-3 Organs and Tissue Distribution

**Figure 1:**
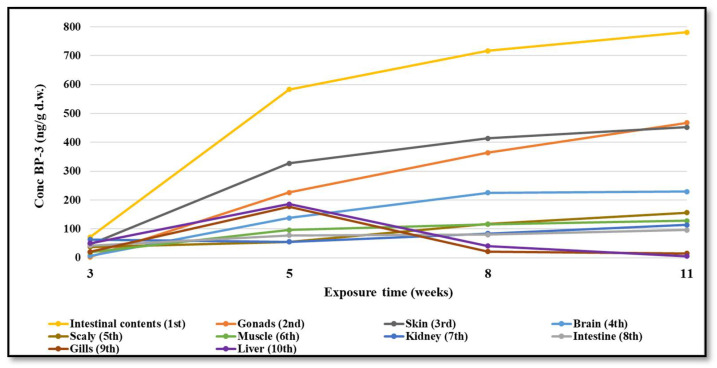
BP-3 concentration values detected in tissue and organs during the bioconcentration experiments.

Biological samples (brain, gonad, intestine and intestinal contents, kidneys, muscles, liver, gills, skin and scaly) were analyzed both to determine the concentration of BP-3 accumulated and to identify and quantify its biotransformation products. In the biological samples taken from the test aquarium at time t0 and in all fish samples taken from the control aquarium, no BP-3 was detected. By contrast, three weeks after the experiment started, BP-3 was found in all organs of the test fish in concentrations ranging from 3.2 to 782 ng/g d.w. An accumulation of BP-3 over time was observed in almost all organs, especially in the gonad, skin, brain and scaly (Figure 1, and the results expressed as averages of 3 replicates ± SD are given in Table S7). However, the highest concentration of BP-3 was determined in the intestinal contents, reaching up to 782 ng/g d.w. The fact that the highest measured levels of BP-3 were in the skin and in the intestinal contents suggest the two main ways in which the organic compound reaches the fish’s body: by ingestion and absorption into the skin. Dermal exposure and ingestion were also reported for humans [36]. Concentrations similar to those determined in the skin have been observed in the gonad. The accumulation of the organic compound in this organ is dangerous in view of the endocrine disrupting effects of this compound that are well known [37].

In the muscles, kidneys, intestines and scaly, a slower increase in BP-3 concentration was observed over time, the maxima being in the range of 96–156 ng/g d.w. Unlike the rest of the organs, the concentration of BP-3 in the liver and gills increased in weeks 3 and 5, reaching up to 186 ng/g d.w in the liver and 177 ng/g d.w. in the gills, after which there was a decrease in weeks 8 and 11, the measured values reaching up to 6 ng/g d.w. in the liver and 15 ng/g d.w. in the gills. For these two organs, we can assume that the decrease in BP-3 concentration is due to an accentuated metabolism of BP-3.

Given that no studies have been reported in the literature on in vivo bioaccumulation experiments of BP-3 in fish organs, but only in situ monitoring, the concentrations determined in this study are higher than those published in the literature [18,38,39,40]. This difference is to be expected considering the use of a higher BP-3 test concentration than the actual concentration present in surface waters and keeping it constant throughout the experiment, in order to observe in a relatively short time the bioaccumulation and biotransformation of BP-3 in the fish body.

### 2.6. Bioconcentration Factors of BP-3

The bioconcentration factors (BCF) were calculated taking into account the BP-3 concentrations found in the organs in the sampling campaigns and the average BP-3 concentrations in water measured over the exposure periods of 3, 5, 9 and 11 weeks. Values of BCF higher than 1 means that BP-3 can easily bioaccumulate in organs and tissue, while values lower than 1 means no bioaccumulation process is involved [41]. All organs were predisposed to BP-3 bioaccumulation. A variety of BCFs were determined depending on the target organ, with the highest average values being in the brain, gonad and skin (Table 3).

The first 3 weeks of exposure showed BCFs in the range of 0.18–3.59 with a potential for bioaccumulation higher than 1 in most organs except the brain and gonad. After 5 weeks, a maximum potential for bioaccumulation of BP-3 (8.30–49.10) was recorded for all organs analyzed in the following order: skin, gonad, liver, gills, brain, muscles, intestine, kidneys. In the intestinal contents, the BCF was 87.4 ± 17.5, indicating that BP-3 is taken from contaminated media into the digestive system. After 9 weeks, a drastic decrease (≥50%) in the potential for bioaccumulation was observed in all organs. The values calculated in the period 9–11 weeks were maintained, highlighting a phase of balance and a phenomenon of saturation of the body in the accumulation of BP-3. During this period, constant BP-3 (<10%) was observed in the gonad, intestine, kidneys and muscles. During the experiment, an average BP-3 organ-specific BCF of 6.92 was recorded, indicating a high potential for bioaccumulation in fish tissues, especially in the brain and gonad (an finding confirmed by specialized studies on the effects on reproduction [18,38,39,40]). The lower BCF values were determined for liver and gills, these two organs being responsible for the BP-3 metabolization.

### 2.7. BP-3 Metabolites in Fish Organs and Tissue

Biotransformation products of BP-3 were detected in all fish organs and tissue (Figure S4), and the results expressed as averages of 3 replicates ± SD reported (Table S7). The main biotransformation product identified in the gills was 234-HBP. Similarly, to BP-3, the maximum accumulated concentration was determined to be in the samples taken at 5 weeks (177 ng/g d.w.), after which it started to decrease, reaching only 15 ng/g d.w. at the end of the experiment. This decrease coincided with the decrease in the concentration of BP-3 accumulated in the gills and the formation of three other biotransformation products, namely BP-10 (25.5 ng/g d.w.), BP-8 (0.9 ng/g d.w.) and BP-1 (7.9 ng/g d.w.). It is known that BP-3 can be metabolized to three major metabolites BP-1, BP-8 and 234-HBP. It seems that persistence of BP-1 is higher than of BP3 and that it can exhibit estrogenic effects [42].

The major organ of BP-3 biotransformation has been shown to be the liver. In the liver, the main biotransformation products of BP-3 were identified as BP-1, 234-HBP and BP-8. Similarly, to the behavior of the parent compound, the concentration of the degradation products also increased during the first two sample periods, after which a decrease in concentration was observed, being similarly metabolized in the liver. The tri-hydroxylated derivative was displayed the highest concentration, recording a maximum of 345 ng/g d.w. and decreasing to 76 ng/g d.w. at the end of the experiment. BP-1 was the second major biotransformation product. The maximum was reached at 5 weeks, up to 54 ng/g d.w. and decreased to 1 ng/g d.w. in week 11. In very low concentrations, the metabolite BP-8 was also identified in week 5, its concentration being no longer measurable in the last two sampling campaigns. The level of BP-3 accumulated in the muscles and kidneys was very similar (up to 128 ng/g d.w. in the muscles and up to 114 ng/g d.w. in the kidneys). Two biotransformation products were identified in both organs, namely 234-HBP and BP-1. In muscles, the concentration of 234-HBP reached a maximum of 369 ng/g d.w. at the end of the experiment, while the concentration of BP-1 showed a relatively low increase, this being in the range of 4–10 ng/g d.w. In the kidneys, in samples taken at weeks 3 and 5, the concentration value of 234-HBP was up to 296 ng/g d.w., after which the compound was formed in very large quantities, reaching up to 1193 ng/g d.w. at the end of the experiment.

The degree of bioaccumulation of BP-3 in the brain reached up to 229 ng/g d.w. At the level of this organ, two biotransformation products of the parent compound could be identified: 234-HBP and BP-1. The two products were quantified in all samples. Interestingly, at week 5 it can be seen that while the concentration of 234-HBP decreases, the concentration of BP-1 increases (reaching a maximum of 45 ng/g d.w.), this being the result of hydroxylation–dihydroxylation processes. In addition to the high concentration of BP-3 accumulated in the gonad, three biotransformation products were identified and quantified, namely 234-HBP, BP-1, 4-HBP and BP-8. BP-3 undergoes transformations in this organ, the tri-hydroxylated biotransformation product being determined in extremely high concentration of up to 955 ng/g d.w. The di-hydroxylated product, BP-1, was found in all samples analyzed, in concentrations between 17 and 26 ng/g d.w., while the formation of the products 4-HBP and BP-8 was observed only in the samples collected in week 5.

In the intestine, the formation of four biodegradation products was observed. Similarly, to the other organs, the most abundant biotransformation product was the tri-hydroxylated derivative, its concentration increasing over time reaching 1206 ng/g d.w. in week 11. The di-hydroxylated derivative, BP-1, was in second place in abundance. Its concentration also increased over time, reaching up to 75 ng/g d.w. The formation of BP-10 was also observed from week 3 onwards. The concentration of this compound continued to increase over time, but much more slowly compared to its di- and tri-hydroxylated counterparts, reaching a maximum of 13 ng/g d.w. BP-8 was also determined at very low concentrations, but only in samples taken at weeks 3 and 5.

Five biotransformation products were identified in the intestinal contents: 234-HBP, BP-1, BP-10, 4-HBP and BP-8. Given that the highest concentration of BP-3 was determined in the intestinal contents, the transformation products were also identified in much higher concentrations compared to those determined in other organs. The transformation product 234-HBP was found in extremely high concentrations, reaching up to 7172 ng/g d.w. The values determined for BP-1 are the highest compared to the values observed in the other organs, the maximum value being reached in week 11 (1281 ng/g d.w.). BP-10 ranked third in the list of the most abundant biotransformation products identified in the intestinal contents. The determined BP-10 content was between 292 and 363 ng/g d.w. throughout the four samples. BP-8 was determined only in samples taken at week 3 (40 ng/g d.w.), while the concentration of 4-HBP was relatively constant during the experiment (0.7–1 ng/g d.w.).

**Figure 2:**
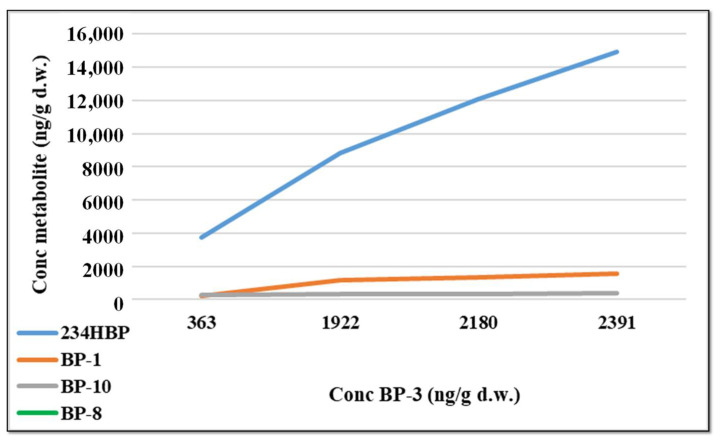
Variation in concentration of identified metabolites compared to the evolution of the BP-3 concentration, in fish.

In the skin, two biotransformation products 234-HBP and BP-1 were identified and quantified. The biotransformation product 234-HBP proved to be the most abundant biotransformation product, its concentration increasing over time and reaching up to 2113 ng/g d.w. The second product, BP-1, also increased over time, but much slower compared to 234-HBP, reaching a maximum of 9.4 ng/g d.w. In scaly, an increase in the concentration of BP-3 and the formation of the tri-hydroxylate product were observed over time. The concentration on the surface of the scaly was much lower (reaching up to 72 ng/g d.w.) than that inside the main organs of the fish. After 5 weeks from the beginning of the experiment, the concentration of BP-3 shows a slower increase, while at the same time a more accentuated increase in the metabolites formed in the organs is observed (Figure 2). As the amount of BP-3 accumulating in the organs approaches equilibrium, a higher percentage of the compound is metabolized in the tissues.

**Figure 3:**
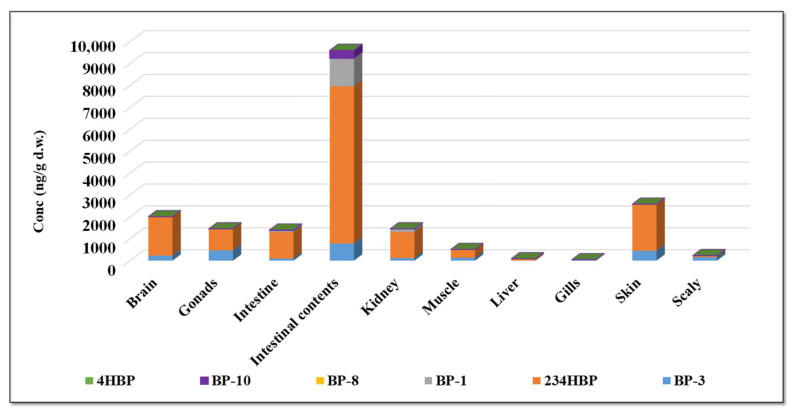
Distribution of BP-3 UV filter and its metabolites in fish organs.

The highest concentrations, both of BP-3 and of metabolites, were found in the intestinal contents (Figure 3), the ingestion of the organic compounds being the main way by which they reach the inside of the fish. The next two organs in which high levels of BP-3 and metabolites were also identified are the brain and skin, but at a much lower level compared to the amounts determined in the intestinal contents.

As can be seen in Figure S5, the major metabolite in all organs was found to be the tri-hydroxylated derivative, 234-HBP, followed by the di-hydroxylated derivative, BP-1, the latter being found in a much lower percentage compared to 234-HBP. 

A very recent study reported that benzophenone derivatives such as 2-hydroxybenzophenone, benzophenone, 4-methylbenzophenone and methyl-2-benzoylbenzoate are ubiquitous in commercial fish products. The average concentration of seven benzophenone derivatives in fresh and salt water fish were 46.4 ng/g and 25.0 ng/g respectively, levels that could induce a health risk due to endocrine disruptor activity especially in the case of infants [43].

### 2.8. BP-3 Metabolites in Water

**Figure 4:**
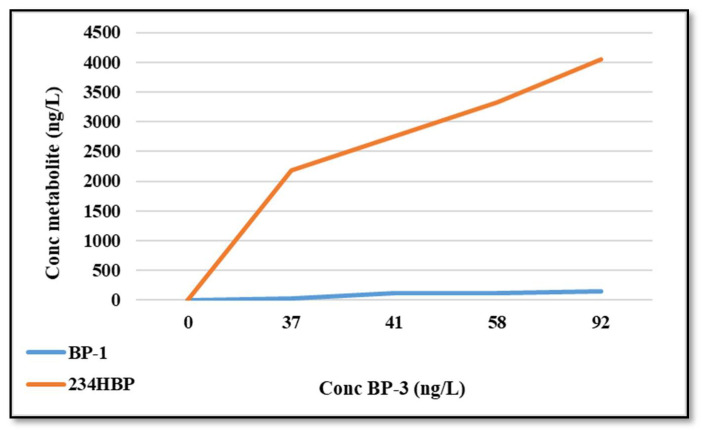
Variation in concentration of identified metabolites compared to the evolution of the BP-3 concentration, in water.

In the water taken with the biological samples (before feeding the fish), a concentration of BP-3 between 37 and 58 µg/L was measured, stabilizing at the end of the experiment at 92 µg/L. Two BP-3 biotransformation products were identified and quantified: 234-HBP and BP-1 (Figure 4). The increase in the concentrations of the two products over time may be the result of their elimination from the body of fish in the form of metabolites.

The total BP-3 concentration added daily in the test pools amounts to 3742 µg/L. The sum of the BP-3 concentration accumulated in the fish organs taken at the end of the experiment is 2447 ng/g d.w. of fish. The difference between the two concentrations is given by the transformation of BP-3 into metabolites in the organs. The concentration values expressed as averages of 3 replicates ± SD are given in Table S8.

### 2.9. Relationships between BP-3 and Corresponding Metabolites in Fish Sample

Strong correlations were observed in gills, between BP-3 and the biotransformation products BP-1 (r = 0.789, *p* ≤ 0.022) and BP-10 (r = 0.724, *p* ≤ 0.016), indicating that BP-3 is directly responsible for the formation of both metabolites (Table S9). Strong positive correlation was also calculated for BP-1 and BP-8 (r = 0.816, *p* ≤ 0.018), suggesting the same source of formation. BP-3 content was highly correlated with 234-HBP (r = 1.000, *p* ≤ 0.001) suggesting that BP-3 is the parent compound of the tri-hydroxylated derivative. Also, BP-8 concentration values determined in gills were highly correlated with BP-10 values (r = 1.000, *p* ≤ 0.001), indicating a common source of formation, namely BP-3.

In liver, strong correlations were observed between BP-3 and BP-8 (r = 0.775, *p* ≤ 0.025), indicating that BP-8 was obtained directly from the BP-3 metabolism process (Table S10). Very strong correlations were determined between BP-3/234-HBP and BP-3/BP-1 (r = 1.000, *p* ≤ 0.001 in both cases), but also between 234-HBP and BP-1 (r = 1.000, *p* ≤ 0.001), suggesting that BP-3 was directly metabolized in 234-HBP and BP-1, but also that the tri-hydroxylated derivatives were obtained from BP-1 metabolization.

In muscle and kidney, strong positive correlations were observed both between BP-3/234-HBP and BP-3/BP-1 and between 234-HBP and BP-1 (r = 1.000, *p* ≤ 0.001) (Tables S11 and S12). Thus, it can be concluded that BP-3 is the parent compound for both bi- and tri-hydroxylated derivatives, but also that BP-1 could be metabolized to 234-HBP.

BP-3 concentration values determined in brain tissues were correlated with the biotransformation product 234-HBP, being the most abundant metabolite detected in this organ (Table S13). In gonads, strong relationships were observed between the pairs: BP-3/234-HBP, BP-3/BP-1 and 234-HBP/BP-1, suggesting that the two compounds 234-HBP and BP-1 are directly obtained from BP-3 metabolization, while the relationship between 4-HBP and BP-8 is indicative of a secondary common source of metabolization (Table S14).

In intestine and intestinal content, Pearson’s correlation test showed a high correlation between BP-3/234-HBP, BP-3/BP-1, BP-3/BP-10, 234-HBP/BP-1, 234-HBP/BP-10 and BP-1/BP10 (*p* ≤ 0.05) (Tables S15 and S16). The results also indicate strong relationships between BP-3 and its biotransformation products. Strong positive correlations were observed between BP-3/BP-1, 234-HBP/BP-1 and 234-HBP/BP-8 (*p* ≤ 0.05) in skin, while BP-3 levels from skin and scaly were highly correlated with 234-HBP values (r = 1.000, *p* ≤ 0.001) (Tables S17 and S18).

### 2.10. BP-3 Metabolization Pathway in Fish Organs

**Figure 5:**
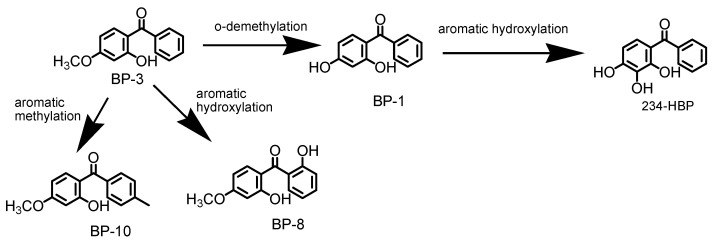
BP-3 metabolization pathway in fish organs.

BP-3 bioaccumulated in the organs of fish can be hydroxylated to form metabolites such as: BP-1, 234-HBP and BP-8 (Figure 5). BP-1 can be obtained by demethylation of the methoxy group (o-demethylation) from the first aromatic ring of BP-3, while BP-8 can be formed by aromatic hydroxylation of the second aromatic nucleus of BP-3 in ortho position. The metabolite 234-HBP can also be formed by the aromatic hydroxylation of the first aromatic nucleus of BP-1. Similar metabolic compounds of BP-3 were also reported in similar studies [10,44,45].

Freshwater fish production in Europe was estimated at 20% of total fish production and carp represent one of the most important sources of food for humans especially in Eastern parts of Europe including Romania. Water quality is more and more affected by chemical pollution which implicitly represents a great risk to the quality of this natural food source. Due to human use of BP-3 (especially as an ingredient in cosmetics) and to human consumption of contaminated fish, some recent studies have found related concentrations of oxybenzone in human urine. The percentage of incidence is high at 97%, and this is a great concern for human health [46]. Through extrapolation from vertebrate studies (fish and rats) to humans, the effects of BP-3 could be bioaccumulation and endocrine disruption. In addition, other studies have reported more severe BP-3 effects such as modification in mRNA and protein expression levels associated with apoptosis and neurotoxicity [47].

## 3. Materials and Methods

### 3.1. Chemicals and Materials

Analytical standard 2-hydroxy-4-methoxy-benzophenone (BP-3), and metabolites: 4-hydroxybenzophenone (4-HBP), 2,4-dihydroxybenzophenone (BP-1), 2,2′,4,4′-tetrahydroxybenzophenone (BP-2), 2,2′-dihydroxy-4-methoxy-benzophenone (BP-8), 2,3,4-trihydroxybenzophenone (234-HBP), and 2-hydroxy-4-methoxy-4′-methyl-benzophenone (BP-10) were purchased from Sigma-Aldrich (Darmstadt, Germany). Isotope-labeled benzophenone-^13^C (^13^C-BP), formic acid and silica gel were also acquired from Sigma-Aldrich (Darmstadt, Germany). Methanol and acetonitrile were provided by Merck (Darmstadt, Germany), while ultrapure water was prepared in-house using the Millipore Milli-Q purification system (Millipore, Darmstadt, Germany).

### 3.2. LC-MS/MS Analytical Methods

Analytical experiments were performed using a liquid-chromatograph system (Agilent 1260, Agilent Technologies, Waldbronn, Germany) coupled with a triple quadrupole mass spectrometer (Agilent 6410B, Agilent Technologies, Waldbronn, Germany), with an ESI operated in both negative and positive modes.

BP-3 concentration in water was monitored by direct injection of samples using the LC-MS/MS system. A volume of 2 µL of water was injected directly into a Luna C18 chromatographic column (150 mm × 2.0 mm, 3.0 µm, Phenomenex, Aschaffenburg, Germany), maintained at a constant temperature of 30 °C. BP-3 was eluted from the chromatographic column using a mobile phase consisting of 0.15% formic acid (A) and acetonitrile (B), 20:80 (*v*/*v*), at a flow rate of 0.2 mL/min. Electrospray ionization source was used in positive mode. MS/MS detection was performed in MRM (multiple reaction monitoring) mode (*m*/*z*: 229 > 151), in less than 4.5 min.

Determination of BP-3 and its metabolites in fish tissue and organs were also determined using the MRM acquisition mode and 1 µL injection volume. The same hydrophobic Luna C18 chromatographic column and the mobile phase 0.1% AF (A) and methanol (B) at a flow rate of 0.2 mL/min were used to separate the target compounds, similarly to the method used for the determination of BP-3 in water. Elution of BP-3 and the six metabolites was performed using an elution gradient, which consisted of increasing the percentage of B from 5 to 95% in 3 min, maintaining this percentage for 7 min and returning to 55% B, with a stop time of 20 min for column rebalancing. The MRM transitions for all analytes and the values of the MS parameters are presented in Table S1. ESI parameters were: capillary voltage (5000 V), drying gas temperature (300 °C), drying gas flow (9 L/min) and nebulizer pressure (40 psi). To increase the determination sensitivity, the detector acquisition was set with seven time segments (Table S2).

### 3.3. Quality Assurance/Quality Control

The LC-MS/MS method for water samples provided quantification limits in the range 0.2–1.2 ng/L (Table S3). In fish samples (muscle) the quantification limits ranged from 0.12 to 0.78 ng/g d.w. The analytical recoveries were situated between 74% and 115%. Matrix effects (ME %) were calculated using the following equation:

ME (%) = (A_spiked matrix_/A_standard solution)_ × 100 (1)

where A_spiked matrix_ represents the pick area of the analytes spiked in the matrix (muscle) and A_standard solution_ is the pick area of the analytes dissolved in mobile phase. ME (%) = 100%—matrix effect does not exist; ME (%) < 100%—ion suppression; ME (%) > 100%—ion enhancement. The obtained values are given in Table S4. In order to calculate the real concentration of the analytes in biological samples, both the recovery and the matrix effects values were taken into account.

Bioconcentration factors (BCF) of BP-3 in fish organs and tissue were determined as the ratio between the concentration of BP-3 in fish organs and tissue (C, ng/kg d.w.) under equilibrium condition divided by the concentration of the BP-3 in water (Cw, µg/L), as shown in Equation (2) [48].

BCF = C/Cw(2)

### 3.4. Laboratory Conditions

#### 3.4.1. Exposure Condition

All experiments were performed in chlorine-free tap water named dilution water. The biological material used in the bioconcentration experiments was fish—European carp (*Cyprinus carpio*), weighing approximately 25 g/individual.

*Cyprinus carpio* is known as the common carp and is present in most rivers and lakes in Europe and Asia, so these vertebrate species can serve as a biomonitoring tool to assess the presence of BP-3 in water resources. Also, this fish has an ecological and economic importance, being at a considerable level in the food chain and aquatic biodiversity and on the other hand a significant source of food for humans.

The fish species used is often used by laboratories in biotests of acute and chronic lethal toxicity, presenting ease for testing and relative sensitivity to potentially dangerous chemicals. Fish were purchased in April 2019 from the Fish Culture Research and Development Station NUCET, Romania. Subsequent to their acquisition, the fish were acclimatized to laboratory conditions for three weeks within the Aquatic Biobase of INCD ECOIND. Fish maintenance and experiments were performed in accordance with the Guide for the Use and Maintenance of Laboratory Animals. The experimental studies were performed according to Organization for Economic Co-operation and Development (OECD) recommendations and supervised by the Commission of Ethics and Professional Deontology of INCD ECOIND (Internal Procedure no. 2555/17.02.2021). The testing procedure was in accordance with OECD 305: Bioaccumulation in fish: aqueous and dietary exposure (2012) [49].

The bioconcentration assay was conducted at 100 μg/L of BP-3 for 11 weeks to measure BP-3 accumulation and possible biotransformation in organs and tissue of *Cyprinus carpio*. For a good solubility the BP-3 stock solutions were prepared in ethanol 96%. Suitable amounts of stock solution were homogenized in dilution water to prepare the testing solutions for fish exposure.

The concentration of BP-3 in *Cyprinus carpio*, experimentally exposed to BP-3, was assessed as follows: initially the fish were measured and weighed to ensure homogeneous lots, 25 specimens selected for each test experiment and control experiment. The characteristics of fish specimens are given in Tables S5 and S6. The control experiment was similar to the experimental test, except that it did not contain the test chemical. The tests were carried out in 100 L glass aquariums which allowed for 80 L of prepared test solution, at a constant temperature of 20 ± 4 °C, with permanent aeration and a light exposure of 12–16 h. The test was performed in a semi-static system with the change of test solutions every 48 h (except on weekends). The fish were fed every morning with an amount equivalent to 1% of the tested group weight. The food used was AquaPond sticks (Valman, Castelnovo, Italy) based on cereals, vegetable extracts, yeasts, vitamins and other minerals. The feed ration was recalculated after each biological sampling campaign. There was no mortality during the experimentation period and no deviant behaviors or any visible toxic symptoms.

Biological samples (control and test) were taken at t_0_ (before contamination) and at 3, 5, 8 and 11 weeks, respectively, from the beginning of the experiment. At each sampling time, 3 fish per control and test were selected, measured and weighed. The fish were sacrificed on the ice through spinal nerve dissection. The fish organs (gills, liver, intestine, kidney, gonads, brain, skin, muscle, scaly, intestinal content) for each of the two categories of samples were homogenized in individual containers per type of tissue. The samples were frozen at −4 °C and then dried by lyophilization at low temperature (−110 °C). After drying, they were shredded/ground, homogenized, weighed and subjected to the ultrasonic-assisted extraction process.

#### 3.4.2. Water Analysis

Before exposure experiments, the quality of the dilution water used for preparing test solution was analyzed for pH, oxygen saturation, temperature, total hardness, organic content, suspensions, free residual chlorine, metals, ammoniumand sulphates, using ISO standardized methods. Also, the testing solutions were periodically monitored for pH, oxygen saturation, temperature, total hardness, organic content and suspensions in order to assure fish survival conditions and the reduction of interferences that may occur in the measurement of BP-3 concentrations due to the presence of suspended organic matter.

In the first 9 days after the start of the experiment, the concentration of BP-3 was monitored three times a day, and by the end of the experiment, the BP-3 concentration was tested twice a day, every day to verify the amount of BP-3 and metabolites.

#### 3.4.3. Biota Analysis

For biota analysis, an extraction technique was modified and adapted for isolation and concentration of BP-3 and metabolites in tissue and organs using ultrasound-assisted extraction technique (EAU) [17].

The extraction was performed in 20 mL glass vials, using 250 mg of solid sample spiked with 1 mL of ^13^C-BP (50 µg/L) and 10 mL of methanol. The mixture was vortexed for 1 min, after which it was subjected to ultrasound extraction for 30 min at a temperature of 30 °C. The supernatant was separated by centrifugation at 3000 rpm for 10 min and the solid sample was subjected to a new extractive process. Finally, the organic phases were combined and purified on silica gel. The extracts were purified using silica gel, evaporated to dryness, dissolved in 1 mL binary mixture of water and acetonitrile 60/40 (*v*/*v*) and transferred to an Eppendorf vial. The samples were stored at 4 °C for two hours, after which they were again centrifuged for 10 min at 14,000× *g* rpm for precipitate decantation. The supernatant was then transferred to an HPLC vial and analyzed by LC-MS/MS. 

#### 3.4.4. Matrix Effects

The matrix effect (ME) is a limitation of the quantitative analysis that affects the reproducibility, linearity and accuracy of the methods. Studies to evaluate the matrix effect have shown that the ESI source is much more influenced by the matrix effect due to its ionization mechanism in which the analyte is ionized in the liquid phase. Improper suppression or improvement of the analytical signal may occur during the entire series of events preceding the analyte access to the MS detector.

In order to determine the effect of the complex matrix of the biological samples in the ionization source of the mass spectrometer detector, the post-extraction addition method was chosen. A number of three muscle samples (250 mg) were subjected to the ultrasound-assisted extraction process. The extracts obtained were contaminated with a known concentration (100 µg/L) of BP-3 and metabolites (BP-2, 234-HBP, 4-HBP, BP-1, BP-8, BP-10) and internal standard in one milliliter of methanol and were analyzed.

The matrix effects were calculated by establishing the ratio between the analytes’ corresponding area in the contaminated sample after UAE (area obtained by subtracting the area corresponding to the analytes from the uncontaminated sample) to the peak area corresponding to a standard solution with identical concentration with which the sample was contaminated, without having been subjected to the SPE extraction procedure. The matrix effects were calculated as the ratio between the analytical signal generated by the analyte in the sample and the signal generated by the analyte in the standard solution, expressed in % ME (Equation (1)).

In assessing the matrix effect for BP-3 and metabolites, a major influence of the biological matrix on the ESI ionization source was observed (Table S4). The detected values generates either a suppression of the analytical signal, this being the case for BP-2, 234-HBP, BP-8 and BP-10, or an erroneous improvement of it, as observed for 4-HBP, BP-1 and BP- 3. For this reason, all the results obtained for the biological samples were corrected with the ME values.

#### 3.4.5. Statistical Analysis

The results of BP-3 and metabolites as concentrations in the control and experimental test were expressed as average (*n* = 3) ± standard deviation (SD) in ng/g d.w. in the case of fish samples and in µg/L in the case of solutions. The correlations in concentration between BP-3 and corresponding metabolite in fish organs and tissue were measured using Pearson’s correlation test (r). The relationships between the parent compound and corresponding biotransformation product were considered significant when *p* ≤ 0.05 and highly significant when *p* ≤ 0.001.

## 4. Conclusions

The bioconcentration of BP-3 in *Cyprinus carpio* was studied in laboratory-controlled experiments in order to assess its BCF values, along with its distribution and that of its identified by-products as measured in brain, gonad, intestine and intestinal contents, kidneys, muscles, liver, gills, skin and scaly.

BP-3 was detected in all organs, accumulated markedly in gonad tissue in concentration up to 468 ng/g d.w, and a maximum concentration value was detected in the intestinal content (782 ng/g d.w.). The present study shows that the highest BCF values were in the range of 8.30 to 49.10 at five weeks of contamination, indicating a high potential for bioaccumulation in fish tissues, especially in the brain and gonad. Five BP-3 by-products were identified, and the majority were detected in organs. The tri-hydroxylated derivative (234-HBP) proved to be the major by-product and was detected in all organs, followed by the di-hydroxylated metabolite (BP-1) and BP-10, detected especially in intestine and intestinal content, and in the case of BP-1, in the kidney also. The study raises concerns for both ecological and human health. The results could be considered in the revision of control regulations for the protection of both environmental and human health.

## Figures and Tables

**Table 1:** Actual concentrations measured in the dilution water without fish after spiking with a nominal concentration of 100 µg/L BP-3.

Time (h)	Measured Concentration (µg/L) ± SD (*n* = 3)
0	100 ± 1.48
24	95.5 ± 1.41
48	97.7 ± 1.45
72	98.2 ± 1.45
96	99.7 ± 1.47
t_120_	98.5 ± 1.46
Average	98.3
CV%	1.48

Note: The results are expressed as averages of 3 replicates ± SD.

**Table 2:** Actual BP-3 concentrations measured in water with fish after spiking with a nominal concentration of 100 µg/L.

Time in Days	t_0_	30 min after Fish Exposure	8:30 a.m.	13:30 p.m. Post Spike	15:30 p.m.
1	99.2 ± 11.9	78.3 ± 9.4	-	-	-
2	-	-	7.2 ± 0.9	79.3 ± 9.5	55.8 ± 8.0
3	-	-	82.9 ± 9.9 *	80.5 ± 9.7	66.6 ± 8.0
4	-	-	9.5 ± 1.1	52.3 ± 6.3	102.5 ± 12.3
5	-	-	26.2 ± 3.1	17.7 ± 2.1	64.4 ± 7.7
6	-	-	92.1 ± 11.1 *	82.8 ± 9.9	49.6 ± 6.0
7	-	-	26.3 ± 3.2	70.3 ± 8.4	50.6 ± 6.1
8	-	-	24.7 ± 3.0	81.8 ± 9.8	55.3 ± 6.6
9	-	-	86.3 ± 10.4 *	73.8 ± 8.9	17.9 ± 2.1

* Concentration determined after changing 80% of the water every 48 h and spiked with a nominal concentration of 100 µg/L. The results are expressed as averages of 3 replicates ± SD.

**Table 3:** BCF values determined in fish samples.

Exposure Time (Weeks, w)	3 w	5 w	9 w	11 w	Average BCF
Intestinal content	4.04 ± 0.80	87.4 ± 17.5	-	-	16.9 ± 3.38
Skin	2.67 ± 0.53	49.1 ± 9.82	17.3 ± 3.46	15.7 ± 3.14	16.1 ± 3.22
Gonads	0.18 ± 0.03	33.9 ± 6.78	15.3 ± 3.05	16.2 ± 3.24	13.7 ± 2.75
Liver	2.84 ± 0.56	27.9 ± 5.58	1.72 ± 0.34	0.20 ± 0.04	3.67 ± 0.73
Gills	1.19 ± 0.23	26.6 ± 5.32	0.91 ± 0.18	0.52 ± 0.10	3.04 ± 0.60
Brain	0.41 ± 0.08	20.7 ± 4.14	9.45 ± 1.89	7.95 ± 1.59	7.78 ± 1.55
Muscle	1.07 ± 0.21	14.4 ± 2.88	4.88 ± 0.97	4.44 ± 0.88	4.66 ± 0.93
Intestine	2.22 ± 0.44	11.6 ± 2.32	3.37 ± 0.67	3.34 ± 0.66	3.81 ± 0.76
Kidney	3.59 ± 0.71	8.30 ± 1.66	3.51 ± 0.70	3.95 ± 0.79	4.11 ± 0.82
Scaly	2.12 ± 0.42	-	4.89 ± 0.97	5.41 ± 1.08	5.37 ± 1.07

Note: The results are expressed as averages of 3 replicates ± SD.

## Data Availability

Data is contained within the article (or Supplementary Material).

## Supplementary Materials

The following supporting information can be downloaded at:https://www.mdpi.com/article/10.3390/foods11111627/s1, Table S1: Experimental condition usede for identification of BP-3 and metabolites in fish tesue and organs; Table S2: Acquisition segments set for BP-3 and metabolite detection; Figure S1: MRM chromatogram obtained for UV-Filters and IS (50 ng/mL); Table S3: Recoveries (R) and limits of quantification (LOQ) obtained for water and fish tissue; Table S4: Matrix effect values determined for BP-3 and metabolites in the biological matrix; Table S5: Initial characteristics of the specimens selected for the bioconcentration test; Table S6: Final characteristics of the specimens selected for the bioconcentration test; Figure S2: BP-3 variation in water aquarium during the bioaccumulation experiment; Figure S3: BP-3 concentration values in tissue and organs; Figure S4: Occurrence of BP-3 biotransformation products in fish organs and tissue; Table S7: Concentration values of BP-3 and major biotransformation products (ng/g d.w.) identified in organs and tissue; Figure S5: Percentage distribution of BP-3 UV filter and its metabolites in fish organs; Table S8: Concentration values of BP-3 and major biotransformation products (µg/L) identified in water; Table S9: Pearson correlation and *p*-values determined between BP-3 and metabolites in gills; Table S10: Pearson correlation and *p*-values determined between BP-3 and metabolites in liver; Table S11: Pearson correlation and *p*-values determined between BP-3 and metabolites in muscle; Table S12: Pearson correlation and *p*-values determined between BP-3 and metabolites in kidney; Table S13: Pearson correlation and *p*-values determined between BP-3 and metabolites in brain; Table S14: Pearson correlation and *p*-values determined between BP-3 and metabolites in gonads; Table S15: Pearson correlation and *p*-values determined between BP-3 and metabolites in intestine; Table S16: Pearson correlation and *p*-values determined between BP-3 and metabolites in the intestinal content; Table S17: Pearson correlation and *p*-values determined between BP-3 and metabolites in skin; Table S18: Pearson correlation and *p*-values determined between BP-3 and metabolites in scaly.
