# Supplementary material for: In Vivo Bioconcentration, Distribution and Metabolization of Benzophenone-3 (BP-3) by Cyprinus carpio (European Carp)
Source: Foods. 2022 May 31;11(11):1627. doi: 10.3390/foods11111627 (PMC9180567; doi:10.3390/foods11111627)
Supplement: Supplementary file 1 [file foods-11-01627-s001.zip › foods-1727298-supplementary.pdf]

**In vivo bioconcentration, distribution and metabolization of benzophenone-3 (BP-3) by *Cyprinus carpio* (European carp)**

Florentina Laura Chiriac, Irina Eugenia Lucaciu, Iuliana Paun, Florinela Pirvu,

Stefania Gheorghe*

**Table S1**. Experimental condition usede for identification of BP-3 and metabolites in fish tesue and organs

| Compound | Chemical structure | $t_R$ | MRM | Fragment or Voltage (V) | Collizion Energy (V) | Cell Accelerated Voltage (V) | Dwell time (msec) | ESI |
|---|---|---|---|---|---|---|---|---|
| 4HBP | 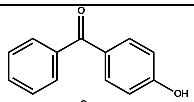 | 3.61 | 197-92 | 150 | 40 | 5 | 165 | Negativ |
| BP-1 | 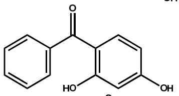 | 4.51 | 213-135 | 130 | 20 | 4 | 250 | Negativ |
| 2,3,4HBP | 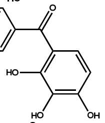 | 3.16 | 229-151 | 135 | 25 | 0 | 165 | Negativ |
| BP-2 | 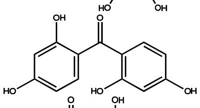 | 2.56 | 245-91 | 110 | 30 | 5 | 165 | Negativ |
| BP-3 | 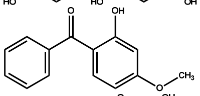 | 8.09 | 229-151 | 135 | 20 | 1 | 250 | Pozitiv |
| BP-8 | 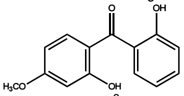 | 5.08 | 245-121 | 150 | 20 | 5 | 250 | Pozitiv |
| BP-10 | 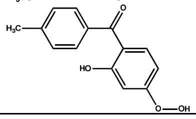 | 8.96 | 243-151 | 130 | 20 | 2 | 250 | Pozitiv |

**Table S2**. Acquisition segments set for BP-3 and metabolite detection.

| Time segment | Start Time (min) | Scan Type | Ionization mode | Div Valve | Store |
|---|---|---|---|---|---|
| 1 | 0 | MRM | ESI | To Waste | No |
| 2 | 2.4 | MRM | ESI | To MS | Yes |
| 3 | 4.3 | MRM | ESI | To MS | Yes |
| 4 | 5.5 | MRM | ESI | To MS | Yes |
| 5 | 7.2 | MRM | ESI | To MS | Yes |
| 6 | 8.8 | MRM | ESI | To MS | Yes |
| 7 | 10 | MRM | ESI | To Waste | No |

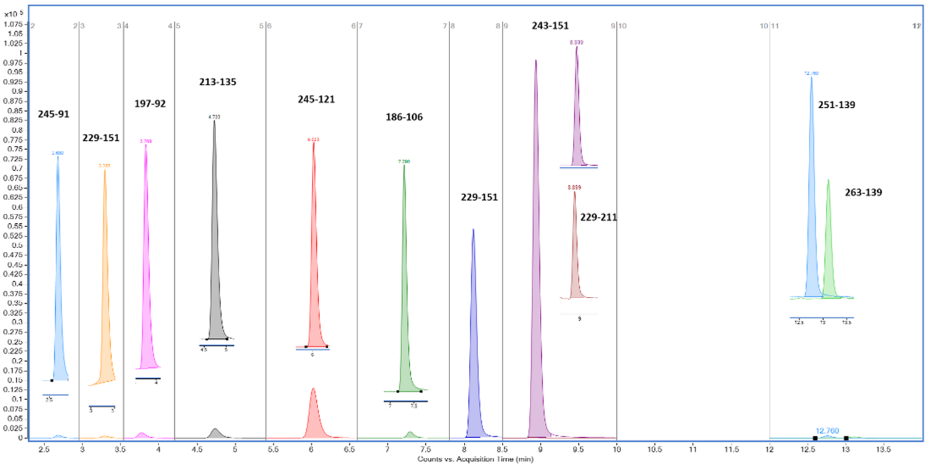


**Figure S1** MRM chromatogram obtained for UV-Filters and IS (50ng/mL)

**Table S3**. Recoveries (R) and limits of quantification (LOQ) obtained for water and fish tissue

| Analytes | R [a] | LOQ [b] | R [c] | LOQ [d] |
|---|---|---|---|---|
| BP-2 | 82.7 | 1.15 | 77.2 | 0.48 |
| 234HBP | 81.4 | 0.92 | 75.4 | 0.12 |
| 4HBP | 90.0 | 0.18 | 88.1 | 0.56 |
| BP-1 | 101.2 | 0.81 | 115 | 0.33 |
| BP-8 | 89.5 | 0.20 | 82.6 | 0.68 |
| BP-3 | 108.7 | 1.22 | 112 | 0.26 |
| BP-10 | 90.2 | 0.41 | 74 | 0.78 |

[a] Recoveries obtained for water (%); [b] LOQ determined for water ng/L; [C] Recoveries obtained for fish tissue (%); [d] LOQ determined for fish tissue in ng/g d.w.

**Table S4**. Matrix effect values determined for BP-3 and metabolites in the biological matrix

| Ananlytes | Matrix effect, % |
|---|---|
| BP-2 | 42 |
| 234-HBP | 70 |
| 4-HBP | 1856 |
| BP-1 | 1791 |
| BP-8 | 78 |
| BP-3 | 3464 |
| BP-10 | 29 |

**Table S5**. Initial characteristics of the specimens selected for the bioconcentration test

| | Test | Martor |
|---|---|---|
| Average weight | 25.47 ± 11.08 g / fish | 24.3 ± 6.28 g/fish |
| Total batch weight | 501 g | 486 g |
| Average length without caudal peduncle | 9.16±1.2 cm | 9.065±0.77 cm |
| Average total length (including caudal peduncle) | 12.078 ±1.41 cm | 11.93 ±0.92 cm |
| Average body height | 3.34±0.57cm | 3.44±0.35 cm |

**Table S6**. Final characteristics of the specimens selected for the bioconcentration test

| | Test | Martor |
|---|---|---|
| Average weight | 27.66 ± 8.17 g / fish | 24.76 ± 6.44 g/fish |
| Total batch weight | 534 g | 493 g |
| Average length without caudal peduncle | 9.5 ± 1.09 cm | 9.4 ± 0.70 cm |
| Average total length (including caudal peduncle) | 12.03 ± 1.34 cm | 12 ± 0.88 cm |
| Average body height | 3.35 ± 0.52 cm | 3.37 ± 0.43 cm |

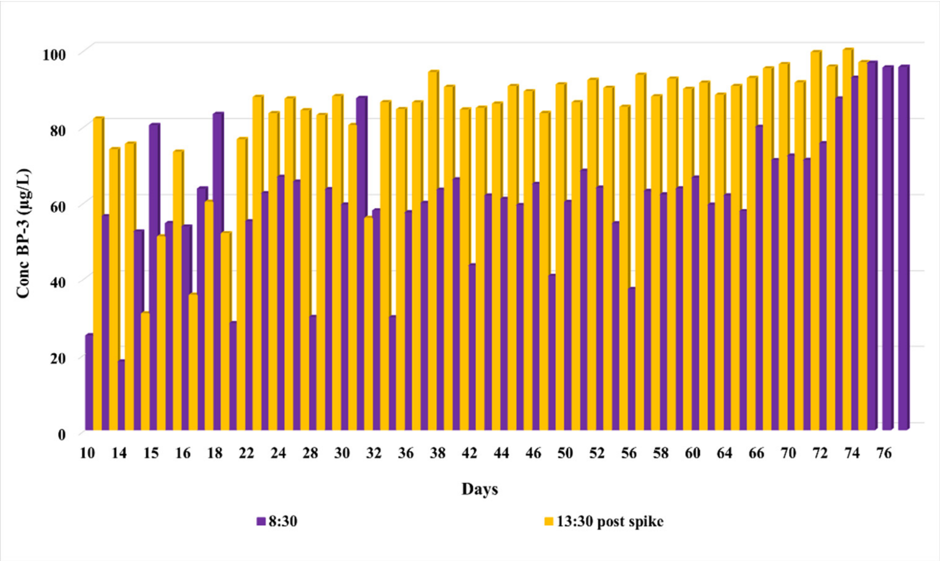


**Figure S2**. BP-3 variation in water aquarium during the bioaccumulation experiment

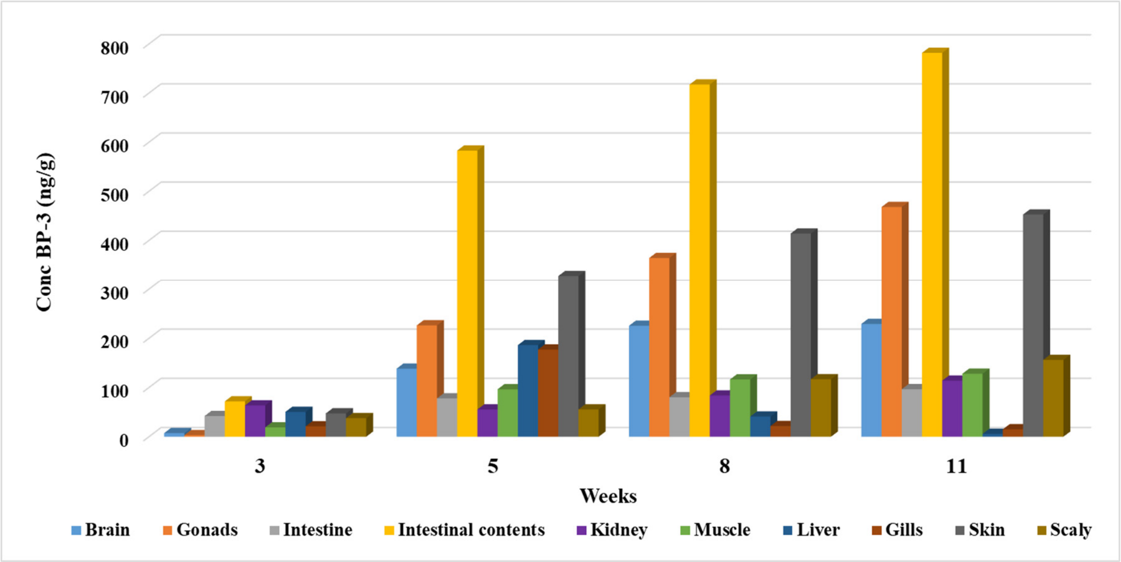


**Figure S3**. BP-3 concentration values in tissue and organs

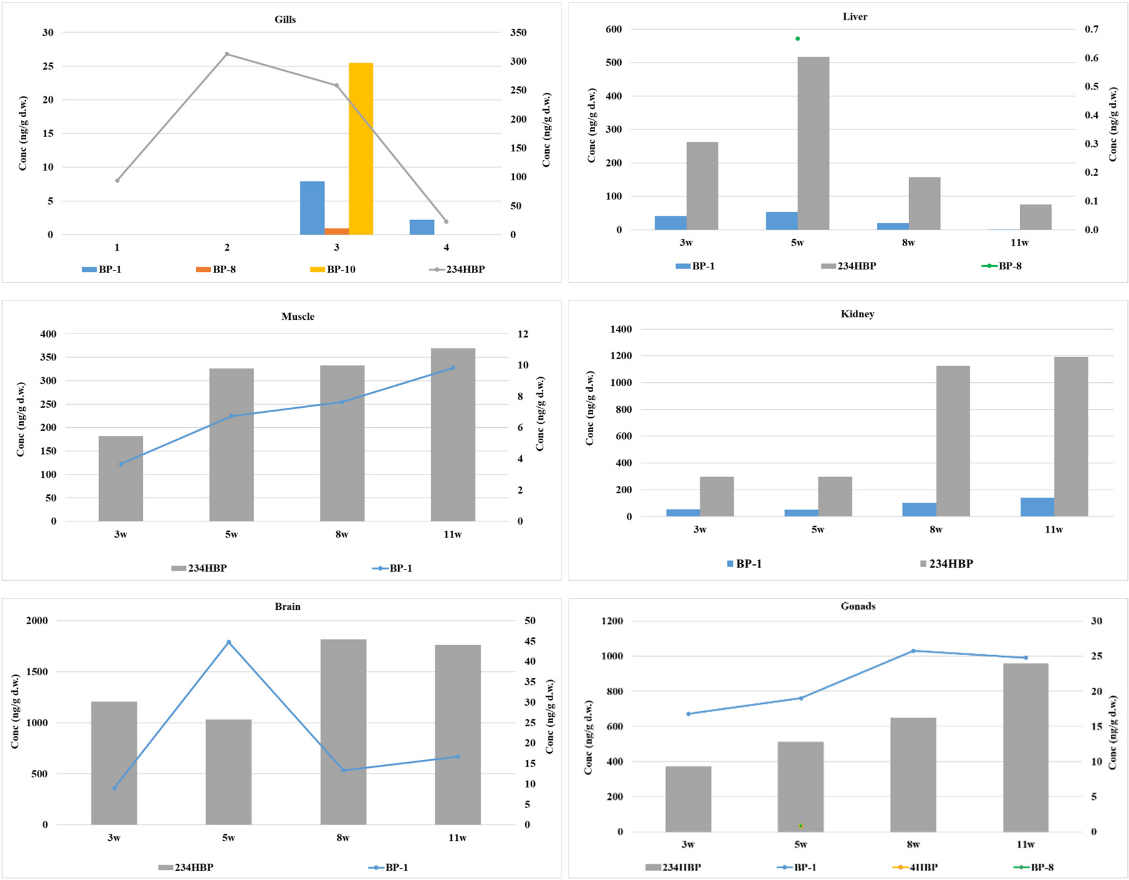

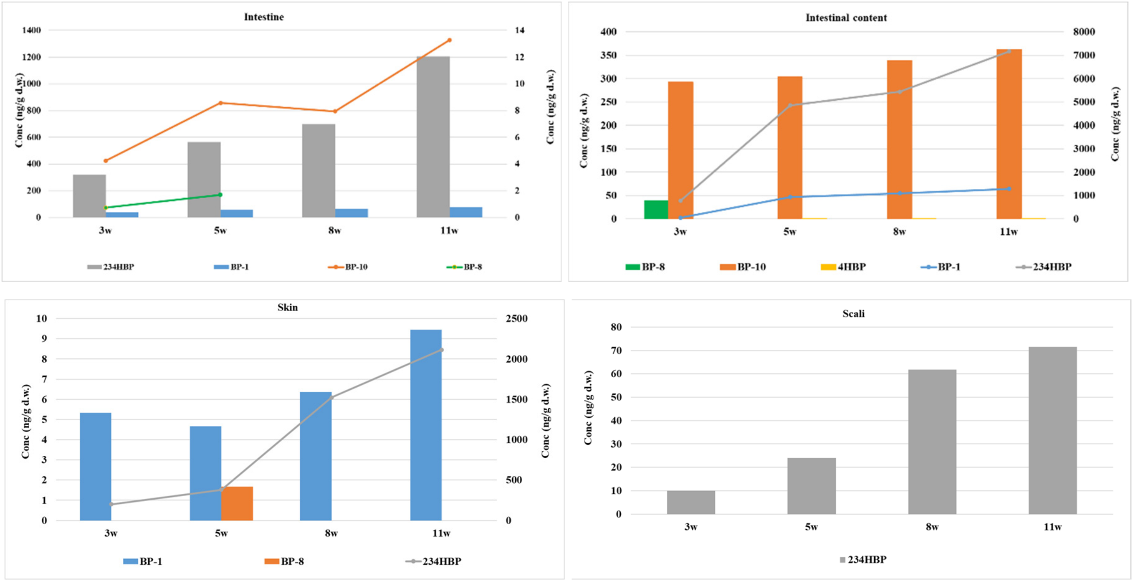


**Figure S4**. Occurrence of BP-3 biotransformation products in fish organs and tissue.

**Table S7**. Concentration values of BP-3 and major biotransformation products (ng/g d.w.) identified in organs and tissue.

| Tissue | Amalytes | 3w | 5w | 8w | 11w |
|---|---|---|---|---|---|
| Brain | 234HBP | 1207±42.2 | 1031±36.1 | 1815±63.5 | 1762±61.7 |
| | BP-1 | 9.01±0.32 | 44.7±1.56 | 13.4±0.47 | 16.7±0.58 |
| | BP-3 | 7.32±0.26 | 138±4.83 | 225±7.88 | 229±8.02 |
| | | | | | |
| Gonads | 234HBP | 369±12.9 | 509±17.8 | 647±22.6 | 955±33.4 |
| | 4HBP | ND | 0.79±0.03 | ND | ND |
| | BP-1 | 16.8±0.59 | 19.0±0.67 | 25.8±0.90 | 24.8±0.87 |
| | BP-8 | ND | 0.83±0.03 | ND | ND |
| | BP-3 | 3.18±0.11 | 226±7.91 | 364±12.7 | 468±16.4 |
| | | | | | |
| Intestine | 234HBP | 321±11.2 | 563±19.7 | 698±24.4 | 1206±42.2 |
| | BP-1 | 38±1.33 | 57.6±2.02 | 65.5±2.29 | 75.3±2.64 |
| | BP-8 | 0.74±0.03 | 1.69±0.06 | ND | ND |
| | BP-3 | 41.9±1.47 | 77.8±2.72 | 80.2±2.81 | 96.5±3.38 |
| | BP-10 | 4.25±0.15 | 8.58±0.30 | 7.95±.28 | 13.3±0.47 |
| | | | | | |
| Intestinal contents | 234HBP | 785±27.5 | 4859±170 | 5442±191 | 7172±251 |
| | 4HBP | ND | 0.72±0.03 | 0.7±0.02 | 0.57±0.02 |
| | BP-1 | 48±1.68 | 931±32.6 | 1097±28.4 | 1281±44.8 |
| | BP-8 | 40±1.40 | ND | ND | ND |
| | BP-3 | 71.8±2.51 | 583±20.4 | 717±25.1 | 782±27.4 |
| | BP-10 | 292±10.2 | 304±10.6 | 339±11.9 | 363±12.7 |
| | | | | | |
| Kidney | 234HBP | 297±10.4 | 296±10.4 | 1126±39.4 | 1193±41.8 |
| | BP-1 | 53.8±1.88 | 51.8±1.81 | 102±3.57 | 142±4.97 |
| | BP-3 | 63.5±2.22 | 55.3±1.94 | 83.7±2.93 | 114±3.99 |

| | | | | | |
|---|---|---|---|---|---|
| Muscle | 234HBP | 182±6.37 | 327±11.4 | 333±11.7 | 369±12.9 |
| | BP-1 | 3.67±0.13 | 6.74±0.24 | 7.64±0.27 | 9.83±0.34 |
| | BP-3 | 19.1±0.67 | 96.3±3.37 | 116±4.06 | 128±4.48 |
| | | | | | |
| Liver | 234HBP | 262±9.17 | 517±17.1 | 157±5.50 | 75.7±2.65 |
| | BP-1 | 40.9±1.43 | 54±1.89 | 20.2±0.71 | 1.08±0.04 |
| | BP-8 | ND | 0.67±0.02 | ND | ND |
| | BP-3 | 50.4±1.76 | 186±6.51 | 40.9±1.43 | 5.72±0.20 |
| | | | | | |
| Gills | 234HBP | 93.7±3.28 | 313±11.0 | 258±9.03 | 22.7±0.79 |
| | BP-1 | ND | ND | 7.90±0.28 | 2.20±0.08 |
| | BP-8 | ND | ND | 0.93±0.03 | ND |
| | BP-3 | 21.1±0.74 | 177±6.20 | 21.6±0.76 | 15.0±0.53 |
| | BP-10 | ND | ND | 25.5±0.89 | ND |
| | | | | | |
| Skin | 234HBP | 199±6.97 | 379±13.3 | 1523±53.3 | 2113±74.0 |
| | BP-1 | 5.33±0.19 | 4.67±0.16 | 6.37±0.22 | 9.45±0.33 |
| | BP-8 | ND | 1.67±0.06 | ND | ND |
| | BP-3 | 47.3±1.66 | 327±11.4 | 414±14.5 | 453±15.9 |
| | | | | | |
| Scaly | 234HBP | 10.0±0.35 | 24.0±0.84 | 61.8±2.16 | 71.5±2.50 |
| | BP-3 | 37.7±1.32 | 55.3±1.94 | 117±4.10 | 156±5.46 |

results expressed as averages of 3 replicates ± SD

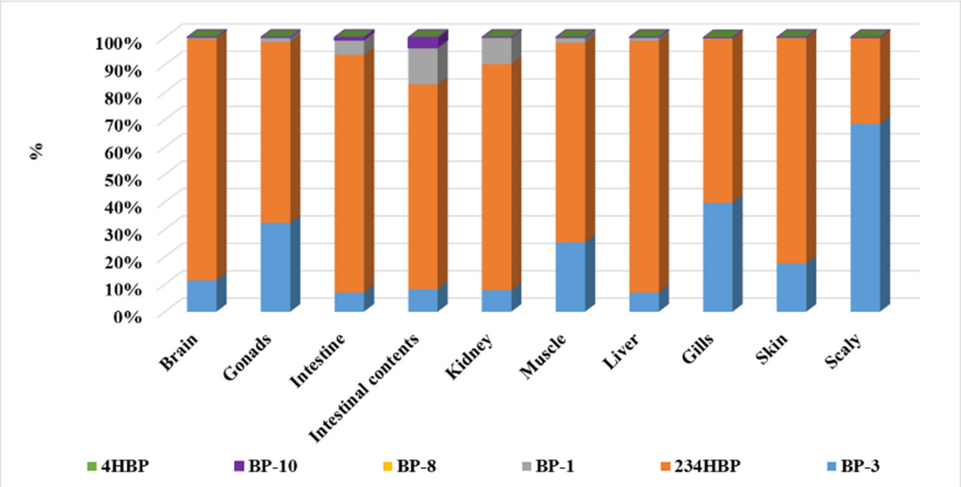


**Figure S5**. Percentage distribution of BP-3 UV filter and its metabolites in fish organs

**Table S8**. Concentration values of BP-3 and major biotransformation products (μg/L) identified in water

| Analytes | 3w | 5w | 8w | 11w |
|---|---|---|---|---|
| 234HBP | 2177±32.2 | 2757±40.8 | 3328±49.3 | 4050±59.5 |
| 4HBP | ND | ND | ND | ND |
| BP-1 | 25±0.37 | 112±1.66 | 125±1.85 | 152±2.25 |
| BP-3 | 371±5.48 | 41±0.61 | 58.3±0.86 | 92.4±1.37 |

results expressed as averages of 3 replicates ± SD

**Table S9**. Pearson correlation and p-values determined between BP-3 and metabolites in gills

| Gills | | BP-1 | BP-8 | BP-3 | BP-10 |
|---|---|---|---|---|---|
| 234HBP | Pearson Corr. | -0.211 | 0.258 | 1.000 | 0.258 |
| | p value | 0.789 | 0.742 | 0.000 | 0.742 |
| BP-1 | Pearson Corr. | | 0.816 | 0.789 | 0.816 |
| | p value | | 0.018 | 0.022 | 0.184 |
| BP-8 | Pearson Corr. | | | 0.258 | 1.000 |
| | p value | | | 0.742 | 0.001 |
| BP-3 | Pearson Corr. | | | | 0.724 |
| | p value | | | | 0.016 |

**Table S10**. Pearson correlation and p-values determined between BP-3 and metabolites in liver

| Liver | | BP-1 | BP-8 | BP-3 |
|---|---|---|---|---|
| 234HBP | Pearson Corr. | 1.000 | 0.775 | 1.000 |
| | p value | 0.000 | 0.225 | 0.000 |
| BP-1 | Pearson Corr. | | 0.775 | 1.000 |
| | p value | | 0.225 | 0.000 |
| BP-8 | Pearson Corr. | | | 0.775 |
| | p value | | | 0.025 |

**Table S11**. Pearson correlation and p-values determined between BP-3 and metabolites in muscle

| Muscle | | BP-1 | BP-3 |
|---|---|---|---|
| 234HBP | Pearson Corr. | 1.000 | 1.000 |
| | p value | 0.000 | 0.000 |
| BP-1 | Pearson Corr. | | 1.000 |
| | p value | | 0.000 |

**Table S12**. Pearson correlation and p-values determined between BP-3 and metabolites in kidney

| Kidney | | BP-1 | BP-3 |
|---|---|---|---|
| 234HBP | Pearson Corr. | 1.000 | 1.000 |
| | p value | 0.000 | 0.000 |
| BP-1 | Pearson Corr. | | 1.000 |
| | p value | | 0.000 |

**Table S13**. Pearson correlation and p-values determined between BP-3 and metabolites in brain

| Brain | | BP-1 | BP-3 |
|---|---|---|---|
| 234HBP | Pearson Corr. | -0.400 | 0.760 |
| | p value | 0.184 | 0.040 |
| BP-1 | Pearson Corr. | | 0.400 |
| | p value | | 0.184 |

**Table S14**. Pearson correlation and p-values determined between BP-3 and metabolites in gonads

| Gonads | | 4HBP | BP-1 | BP-8 | BP-3 |
|---|---|---|---|---|---|
| 234HBP | Pearson Corr. | -0.258 | 0.800 | -0.258 | 1.000 |
| | p value | 0.184 | 0.020 | 0.184 | 0.000 |
| 4-HBP | Pearson Corr. | | -0.258 | 1.000 | -0.258 |
| | p value | | 0.184 | 0.038 | 0.184 |
| BP1 | Pearson Corr. | | | -0.258 | 0.800 |
| | p value | | | 0.184 | 0.020 |
| BP-8 | Pearson Corr. | | | | -0.258 |
| | p value | | | | 0.184 |

**Table S15**. Pearson correlation and p-values determined between BP-3 and metabolites in intestine

| Intestine | | BP-1 | BP-8 | BP-3 | BP-10 |
|---|---|---|---|---|---|
| 234HBP | Pearson Corr. | 1.000 | -0.738 | 1.000 | 0.823 |
| | p value | 0.000 | 0.262 | 0.000 | 0.017 |
| BP-1 | Pearson Corr. | | -0.738 | 1.000 | 0.844 |
| | p value | | 0.262 | 0.000 | 0.025 |
| BP-8 | Pearson Corr. | | | -0.738 | -0.211 |
| | p value | | | 0.262 | -0.714 |
| BP-3 | Pearson Corr. | | | | 0.873 |
| | p value | | | | 0.012 |

**Table S16**. Pearson correlation and p-values determined between BP-3 and metabolites in the intestinal content

| Intestinal content | | 4HBP | BP-1 | BP-8 | BP-3 | BP-10 |
|---|---|---|---|---|---|---|
| 234HBP | Pearson Corr. | 0.200 | 1.000 | -0.775 | 1.000 | 1.000 |
| | p value | 0.800 | 0.000 | 0.225 | 0.000 | 0.000 |
| 4HBP | Pearson Corr. | | 0.200 | -0.775 | 0.200 | 0.200 |
| | p value | | 0.800 | 0.225 | 0.800 | 0.800 |
| BP-1 | Pearson Corr. | | | -0.775 | 1.000 | 1.000 |
| | p value | | | 0.225 | 0.000 | 0.000 |
| BP-8 | Pearson Corr. | | | | -0.844 | -0.775 |
| | p value | | | | 0.025 | 0.225 |
| BP-3 | Pearson Corr. | | | | | 1.000 |
| | p value | | | | | 0.000 |

**Table S17**. Pearson correlation and p-values determined between BP-3 and metabolites in skin

| Skin | | BP-1 | BP-8 | BP-3 |
|---|---|---|---|---|
| 234HBP | Pearson Corr. | 0.800 | 0.000 | 1.000 |
| | p value | 0.020 | 1.000 | 0.000 |
| BP-1 | Pearson Corr. | | -0.775 | 0.800 |
| | p value | | 0.225 | 0.013 |
| BP-8 | Pearson Corr. | | | -0.258 |
| | p value | | | 0.742 |

**Table S18**. Pearson correlation and p-values determined between BP-3 and metabolites in scaly

| Scaly | | BP-3 |
|---|---|---|
| 234HBP | Pearson Corr. | 1.000 |
| | p value | 0.000 |
